# Assessing quality of life among university students: psychometric evidence of the EUROHIS-QOL in a Latin American context

**DOI:** 10.3389/fpubh.2026.1894108

**Published:** 2026-07-15

**Authors:** Carlos Alberto Henao Periañez, Cruz Deicy Jaramillo-Bolívar, Marcio Alexander Castillo-Díaz

**Affiliations:** 1 Escuela de Enfermería, Facultad de Salud, Universidad del Valle, Cali, Colombia; 2 Programa de Enfermería, Facultad de Ciencias de la Salud, Universidad Libre, Cali, Colombia; 3 Departamento de Psicología/Maestría en Psicometría y Evaluación Educativa, Facultad de Ciencias Sociales, Universidad Nacional Autónoma de Honduras, Tegucigalpa, Honduras

**Keywords:** EUROHIS-QOL, measurement invariance, psychometrics, public health, quality of life, university students

## Abstract

**Background:**

Quality of life is an important indicator of wellbeing in university populations, where academic, social, and psychological demands may influence health perceptions. The European Health Interview Survey–Quality of Life Index (EUROHIS-QOL) is a brief quality-of-life instrument derived from the World Health Organization Quality of Life instrument (WHOQOL) framework; however, psychometric evidence in Latin American university students remains limited. This study evaluated the structural validity, reliability, and measurement invariance of the EUROHIS-QOL in Colombian university students through the comparison of alternative factorial models.

**Methods:**

A cross-sectional methodological study was conducted among undergraduate students from a private university in Cali, Colombia (*N* = 614). Confirmatory factor analysis using the weighted least squares mean and variance adjusted estimator (WLSMV) evaluated three competing structures: unidimensional, correlated two-factor, and bifactor models, followed by a theoretically justified model re-specification. Model fit was assessed using the Comparative Fit Index (CFI), Tucker–Lewis Index (TLI), Root Mean Square Error of Approximation (RMSEA), and Standardized Root Mean Square Residual (SRMR). Reliability was examined using ordinal alpha (*α*), McDonald’s omega (*ω*), composite reliability (CR), and average variance extracted (AVE). Measurement invariance across gender, comorbidity status, and employment status was assessed using multigroup CFA.

**Results:**

The original unidimensional and correlated two-factor models demonstrated inadequate fit, whereas the bifactor model failed to converge. A theoretically re-specified unidimensional model including residual covariance between overall quality of life and satisfaction with health demonstrated excellent fit (*χ*^2^ = 44.15, df = 19, CFI = 0.983, TLI = 0.975, RMSEA = 0.046, SRMR = 0.022). Reliability indices were high (*α* = 0.907, *ω* = 0.901, CR = 0.908), and convergent validity was supported (AVE = 0.606). Full measurement invariance was supported across evaluated groups.

**Conclusion:**

The EUROHIS-QOL demonstrated satisfactory psychometric performance in Colombian university students, supporting its use as a brief and reliable measure of quality of life in higher education settings. Findings support an essentially unidimensional representation following theoretically justified model refinement and indicate comparable measurement across relevant sociodemographic groups.

## 1 Introduction

Quality of life is a multidimensional construct widely used to assess individuals’ perceptions of their physical, psychological, social, and environmental wellbeing within the context of their experiences, expectations, and cultural values (1). Over recent decades, the assessment of quality of life has gained increasing importance in public health and health sciences research due to its ability to integrate subjective indicators of wellbeing and functioning beyond traditional measures of morbidity and mortality (2). Furthermore, quality of life is a dynamic concept strongly influenced by contextual, cultural, and social factors, highlighting the need for valid and culturally appropriate instruments to assess this construct across diverse populations (3, 4).

University students represent a particularly relevant population for quality-of-life assessment due to the multiple academic, social, and emotional demands associated with this stage of development (5). Entering and remaining in higher education often entails substantial changes in lifestyle, personal autonomy, social relationships, and academic responsibilities, all of which may significantly influence students’ wellbeing and perceived quality of life (6). Previous research has shown that quality of life among university students is associated with a range of factors, including academic stress, mental health, economic conditions, lifestyle behaviors, and social support (7, 8). Likewise, depression, unhealthy lifestyle patterns, and limited access to institutional support services have been consistently linked to poorer quality of life in this population (9–11). In particular, mental health, sleep quality, physical activity, and academic satisfaction have been identified as significant predictors of quality of life among university students, underscoring the complex and multifactorial nature of this construct within educational settings (8).

In Latin America, the assessment of quality of life among university students is particularly relevant given the social and economic inequalities that may shape both academic experiences and subjective wellbeing during this stage of life (12). However, despite growing interest in this field, psychometric evidence in Latin American university populations remains limited, particularly regarding brief instruments supported by robust validity and reliability evidence that enable culturally appropriate, valid, and comparable assessments. This limitation is especially relevant in population-based and university research settings, where administration time and respondent burden constitute important methodological considerations. Moreover, the diversity of Colombian university populations provides a relevant context for evaluating the applicability of brief quality-of-life measures in Latin America.

The World Health Organization initially developed the World Health Organization Quality of Life instrument (WHOQOL) and subsequently the World Health Organization Quality of Life–Brief version (WHOQOL-BREF) to assess quality of life from a transcultural and multidimensional perspective (1, 13). Although the WHOQOL-BREF has demonstrated adequate psychometric performance across diverse cultural settings, its length may limit feasibility in epidemiological studies and large-scale assessments (14–16). To address the need for shorter and more efficient instruments, the European Health Interview Survey–Quality of Life Index (EUROHIS-QOL) 8-item index was developed as a brief version derived from the WHOQOL-BREF, retaining representative indicators of key quality-of-life domains while substantially reducing respondent burden (17). Previous studies have reported favorable psychometric properties of the EUROHIS-QOL across different populations, including adequate reliability and evidence of structural validity (18–20). Moreover, studies conducted in clinical and community populations have supported its usefulness as a brief and parsimonious measure of quality of life (21, 22).

However, evidence regarding the factorial structure of the EUROHIS-QOL remains inconsistent across studies and cultural contexts. While some studies support an essentially unidimensional structure (17), others have identified alternative factorial solutions or the need to account for residual associations among conceptually overlapping indicators to achieve adequate model fit (18–20). These findings raise questions about the factorial stability and measurement equivalence of the EUROHIS-QOL across different cultural and population contexts and suggest that relationships among global evaluative indicators may not be fully explained by a single latent factor. Despite the increasing use of this instrument, psychometric evidence in Latin American university students remains limited, particularly regarding the comparison of competing factorial models and the assessment of measurement invariance. Consequently, it remains unclear whether the EUROHIS-QOL assesses quality of life in a structurally consistent and comparable manner within this population.

Against this background, a rigorous evaluation of the factorial structure and psychometric properties of the EUROHIS-QOL in Latin American university students is warranted. Accordingly, this study aimed to evaluate the structural validity, reliability, and measurement invariance of the EUROHIS-QOL in Colombian university students through the comparison of alternative factorial models. Assessing measurement invariance is essential to determine whether the instrument evaluates quality of life equivalently across relevant sociodemographic groups, thereby enabling meaningful comparisons between populations.

## 2 Methods

### 2.1 Study design

A cross-sectional methodological study was conducted during the 2025–1 and 2025–2 academic semesters at a private university in Cali, Colombia. The design, conduct, and reporting of the study followed the recommendations of the Strengthening the Reporting of Observational Studies in Epidemiology (STROBE) statement for observational research (23). In addition, the study followed the recommendations of the Consensus-based Standards for the Selection of Health Measurement Instruments (COSMIN) initiative for the evaluation of validity and reliability of health-related measurement instruments (24).

### 2.2 Participants and sampling

The study population comprised undergraduate students enrolled in academic programs from the faculties of Health Sciences, Law and Political and Social Sciences, Economic and Administrative Sciences, and Engineering. During the 2025–1 academic semester, the total undergraduate enrollment across these faculties was 2,909 students, which constituted the target population.

Sample size estimation was conducted using an *a priori* power analysis for structural equation modeling (25). Assuming a small-to-moderate anticipated effect size (0.20), a statistical power of 80%, and a significance level of 5%, the specified model—including two latent variables and eight observed indicators—yielded a minimum required sample size of 223 participants to ensure adequate statistical power and parameter stability.

A proportional stratified sampling strategy was implemented, using academic faculties as strata. The number of participants selected from each faculty was determined proportionally according to student enrollment. Subsequently, eligible students within each stratum were selected through simple random sampling using computer-generated random numbers. To compensate for potential non-response and maintain proportional representation across faculties, additional students were randomly invited following the same procedure.

### 2.3 Eligibility criteria

Students were eligible to participate if they were aged 18 years or older, were officially enrolled in undergraduate programs during the 2025–1 and 2025–2 academic periods, and voluntarily agreed to participate by providing informed consent before completing the survey. Students who declined participation or submitted incomplete questionnaires, particularly in the principal study variables, were excluded from the analysis. Missing data were not imputed, and only fully completed questionnaires meeting all eligibility criteria were retained in the final analytical sample.

### 2.4 Ethical considerations

The study was conducted in accordance with national and institutional ethical standards for research involving human participants. Ethical approval was granted by the Research Ethics and Bioethics Committee of Universidad Libre, Cali Campus (Act No. CAL-131202409-CE, November 8, 2024). The study also adhered to the ethical principles established in Resolution 008430 of 1993 issued by the Colombian Ministry of Health.

Before participation, all students received detailed information regarding the objectives and procedures of the study and provided written informed consent. Participation was voluntary, anonymous, and confidential, and participants were informed of their right to withdraw from the study at any time without academic consequences. The study was classified as minimal risk, and no adverse events were reported during data collection.

### 2.5 Data collection instruments

Data were collected using a structured self-administered online questionnaire developed in Google Forms. The survey included sections assessing sociodemographic and academic characteristics, in addition to the EUROHIS-QOL.

#### 2.5.1 Sociodemographic and academic variables

Sociodemographic and academic information was obtained through a questionnaire specifically designed for this study. The collected variables included age (years), gender, socioeconomic stratum, faculty affiliation, time at university, employment status while studying, and self-reported grade point average (GPA).

Socioeconomic status was categorized according to the Colombian residential stratification system into low, middle, and high strata. Time at university was grouped as less than 2 years and 2 years or more. Employment status was classified according to whether students worked while studying. Academic performance was assessed using self-reported GPA and categorized according to institutional grading ranges.

#### 2.5.2 The EUROHIS-QOL 8-item index

Quality of life was assessed using the EUROHIS-QOL, a brief generic instrument derived from the WHOQOL and the WHOQOL-BREF (1, 14). The EUROHIS-QOL was developed to provide a shorter and less burdensome alternative for epidemiological and population-based studies while preserving the conceptual coverage of the original multidimensional quality of life framework (17). The instrument consists of eight items representing key domains of quality of life, including overall quality of life, satisfaction with health, energy for daily life, financial resources, daily living activities, satisfaction with self, personal relationships, and living conditions. Each item is rated on a five-point Likert-type scale, with higher scores indicating better perceived quality of life (17).

Previous studies have reported adequate psychometric performance of the EUROHIS-QOL across different cultural settings, supporting its reliability and structural validity as a brief measure of quality of life (17, 20). Additionally, cross-cultural studies have included Spanish-speaking populations, supporting the applicability of the instrument in different linguistic and cultural contexts (20).

### 2.6 Statistical analysis

Statistical analyses were conducted using the RStudio environment (version 2025.5.1.513) with R software (version 4.4.2) (26). Confirmatory factor analyses (CFA) were performed using the lavaan package (version 0.6–16) (27), with additional psychometric analyses conducted using the semTools package (version 0.5–6) (28).

**Figure 1:**
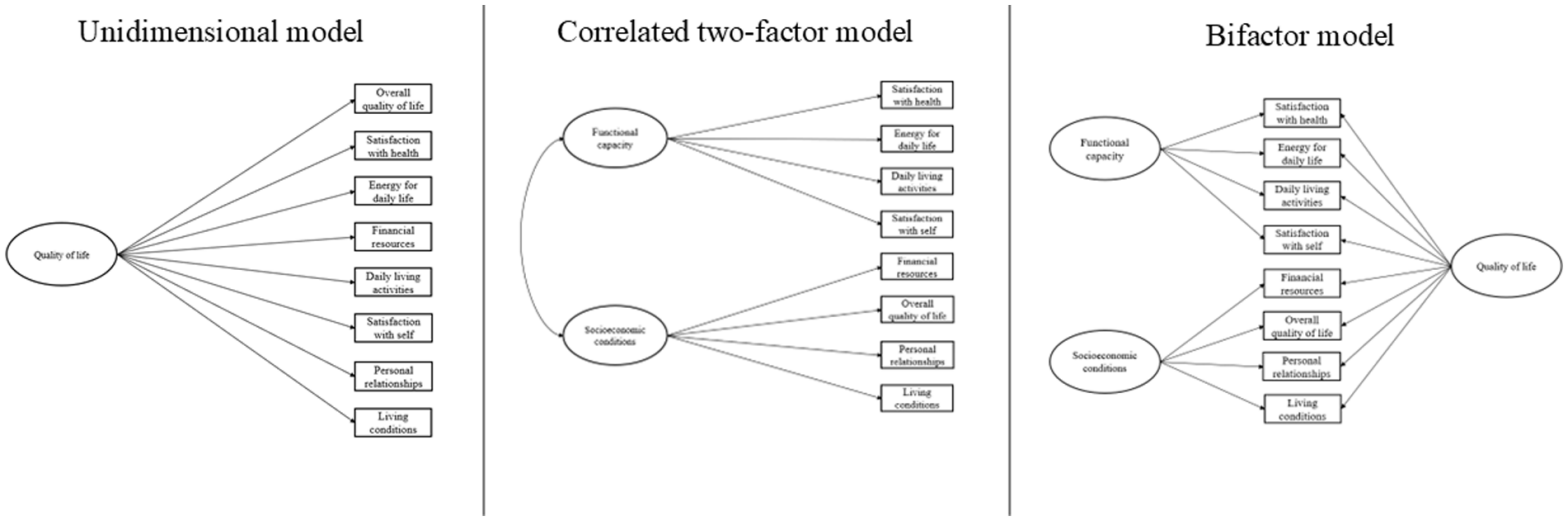
Factorial models evaluated for the EUROHIS-QOL in university students. Model 1 = unidimensional model; Model 2 = correlated two-factor model; Model 3 = bifactor model.

The internal structure of the EUROHIS-QOL was examined through item-level CFA using the weighted least squares mean and variance adjusted (WLSMV) estimator, given the ordinal nature of the response options (29). Three competing factorial models were evaluated: Model 1 specified a unidimensional structure representing a general quality-of-life construct, consistent with the original conceptualization of the instrument (17); Model 2 specified a correlated two-factor structure reflecting functional capacity (satisfaction with health, energy for daily life, daily living activities, and satisfaction with self) and socioeconomic conditions (financial resources, overall quality of life, personal relationships, and living conditions), based on previous psychometric findings in Brazilian adults (19). Model 3 specified a bifactor structure including a general quality-of-life factor and the two specific domains proposed in the correlated model, allowing examination of whether a common latent construct accounted for item covariance beyond domain-specific variance (30). Figure 1 presents the factorial specifications of the competing EUROHIS-QOL models evaluated in the confirmatory factor analyses, including the unidimensional, correlated two-factor, and bifactor structures. Consistent with recommendations for confirmatory factor analysis, model re-specifications were considered only when supported simultaneously by modification indices, theoretical plausibility, and prior conceptual evidence regarding the overlap between global evaluative indicators of quality of life and health status.

Model fit was assessed using multiple goodness-of-fit indices, including the Comparative Fit Index (CFI), Tucker–Lewis Index (TLI), Root Mean Square Error of Approximation (RMSEA), and Standardized Root Mean Square Residual (SRMR). Following commonly recommended criteria, acceptable model fit was considered when CFI and TLI values were ≥ 0.95 and RMSEA and SRMR values were ≤0.08 (31). Competing models were compared considering statistical fit, theoretical interpretability, and parsimony.

Standardized factor loadings (*λ*) were inspected to evaluate the contribution of each item to the latent construct, with values ≥ 0.40 considered acceptable indicators of practical relevance. Internal consistency reliability was assessed using ordinal alpha (*α*) and McDonald’s omega (*ω*), with values ≥ 0.70 interpreted as evidence of satisfactory reliability (32, 33). Convergent validity was examined using the average variance extracted (AVE) and composite reliability (CR), applying commonly recommended thresholds of ≥ 0.50 and ≥ 0.70, respectively (32, 34).

Measurement invariance was evaluated across gender, comorbidity status and working status using multigroup CFA. A hierarchical sequence of nested models was tested, including configural, metric, and scalar invariance. Configural invariance examined whether the factorial structure was equivalent across groups, metric invariance constrained factor loadings to equality, and scalar invariance additionally constrained item thresholds, enabling latent mean comparisons (35). Invariance was considered supported when changes in model fit met recommended criteria (ΔCFI < 0.010 and ΔRMSEA ≤ 0.015) (36). When scalar invariance was established, latent mean differences between groups were estimated by fixing the latent mean of the reference group to zero and freely estimating the comparison group means (37).

## 3 Results

### 3.1 Descriptive analyses

A total of 644 students responded to the survey. Among these, 20 questionnaires (3.1%) were excluded because they contained incomplete data in the primary study variables, while an additional 10 participants (1.6%) were excluded for being younger than 18 years of age. The final sample therefore consisted of 614 university students who fulfilled all eligibility criteria, corresponding to 95.3% of the completed questionnaires and 21.1% of the target population.

The participants had a mean age of 21.62 years (SD = 3.94), with women comprising the majority of the sample (64.17%), and more than half of the students were classified within the middle socioeconomic level (57.98%). The sample distribution across faculties was consistent with the proportional stratified sampling strategy, allowing representation from the principal academic divisions of the university. Regarding academic characteristics, over half of the participants had attended the university for at least 2 years (56.35%), and most indicated that they did not combine study with employment (68.24%). Additional sociodemographic and academic information is summarized in Table 1.

**Table 1:** Sociodemographic and academic characteristics of the study participants (*n* = 614).

Variable	*n* (%)/Mean ± SD
Age (years)	21.62 ± 3.94
Gender	
Female	394 (64.17)
Male	220 (35.83)
Socioeconomic stratum*	
Low	163 (26.55)
Middle	356 (57.98)
High	95 (15.47)
Faculty	
Health Sciences	437 (71.17)
Law, Political and Social Sciences	88 (14.33)
Economic, Administrative and Accounting Sciences	67 (10.91)
Engineering	22 (3.58)
Time at university	
<2 years	268 (43.65)
≥2 years	346 (56.35)
Employment status (study and work)	
No	419 (68.24)
Yes	195 (31.76)
Current grade point average (GPA)	
<3.0	10 (1.63)
3.0–3.9	297 (48.37)
4.0–5.0	276 (44.95)
First semester (not applicable)	31 (5.05)

M = mean; SD = standard deviation. *n* (%) = frequency and percentage. * = The Colombian residential stratification system is widely used as a proxy indicator of socioeconomic conditions; GPA = Grade point average (range: 0.0–5.0).

### 3.2 Item-level descriptive statistics

Item-level descriptive statistics for the EUROHIS-QOL are presented in Table 2. Mean scores ranged from 2.99 (Item 3) to 3.62 (Item 1), indicating moderate levels of perceived quality of life across domains. Standard deviations varied between 0.91 and 1.18, suggesting adequate variability in participant responses. Skewness values ranged from −0.27 to 0.01, while kurtosis values ranged from −0.67 to −0.36, indicating no substantial deviations from univariate normality. Overall, the distributional properties of the items supported their suitability for subsequent confirmatory factor analyses.

**Table 2:** Item-level descriptive statistics for the EUROHIS-QOL items (*N* = 614).

Item	Content domain	*M*	SD	Skewness	Kurtosis
Item 1	Overall quality of life	3.62	0.91	−0.08	−0.64
Item 2	Satisfaction with health	3.32	1.00	0.01	−0.36
Item 3	Energy for daily life	2.99	1.08	−0.07	−0.43
Item 4	Financial resources	3.01	1.09	−0.06	−0.47
Item 5	Daily living activities	3.05	1.09	−0.11	−0.41
Item 6	Satisfaction with self	3.12	1.18	−0.14	−0.67
Item 7	Personal relationships	3.07	1.12	−0.11	−0.49
Item 8	Living conditions	3.31	1.18	−0.27	−0.61

M = mean; SD = standard deviation. Skewness and kurtosis describe distributional properties of each item.

### 3.3 Validity based on internal structure

**Figure 2:**
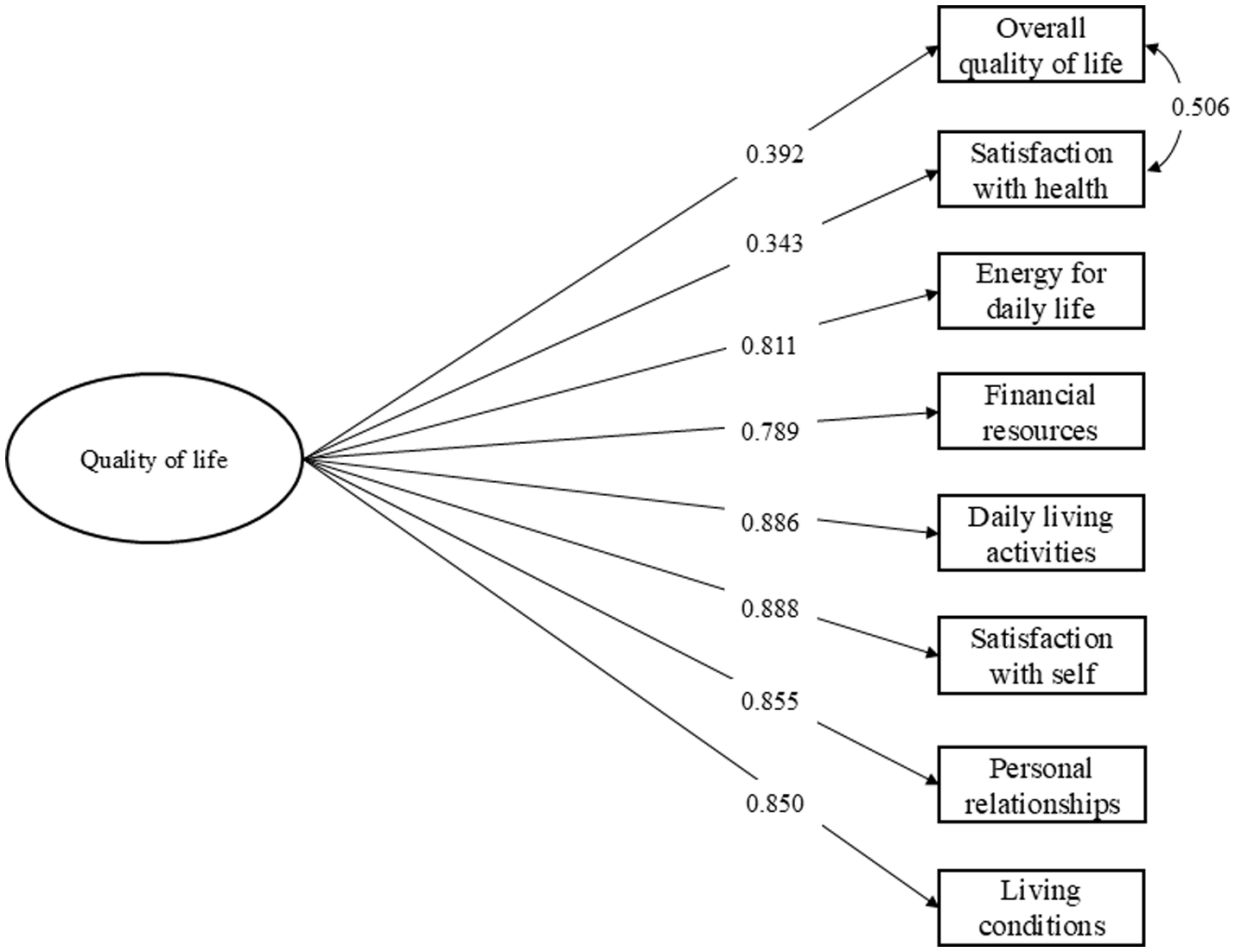
Final re-specified unidimensional EUROHIS-QOL model in Colombian university students. Values represent standardized factor loadings. The curved arrow indicates residual covariance between Item 1 (overall quality of life) and Item 2 (satisfaction with health).

Table 3 presents the fit indices for the factorial models evaluated for the EUROHIS-QOL. The initial unidimensional model demonstrated inadequate fit to the data (*χ*^2^ = 329.379, df = 20, CFI = 0.790, TLI = 0.705, RMSEA = 0.159, SRMR = 0.074). The correlated two-factor model also showed poor fit (*χ*^2^ = 324.317, df = 19, CFI = 0.792, TLI = 0.694, RMSEA = 0.162, SRMR = 0.074). Additionally, a bifactor model was tested, but the solution failed to converge and was therefore not retained. Because Items 1 (“overall quality of life”) and 2 (“satisfaction with health”) represent global evaluative judgments that have been conceptualized as closely related indicators within the WHOQOL framework, residual dependence between these items was considered theoretically plausible. Inspection of modification indices supported this expectation, indicating substantial unexplained covariance between the items (MI = 95.724; EPC = 0.398). Therefore, a residual covariance was specified between these indicators and the re-specified model was subsequently evaluated. This re-specified unidimensional model demonstrated excellent fit to the data (*χ*^2^ = 44.150, df = 19, CFI = 0.983, TLI = 0.975, RMSEA = 0.046, SRMR = 0.022), supporting the essential unidimensionality of the instrument. Figure 2 illustrates the final re-specified unidimensional EUROHIS-QOL model, including the standardized factor loadings and the residual covariance between Item 1 and Item 2.

**Table 3:** Comparison of EUROHIS-QOL factor models in university students.

Model	*χ*^2^	df	CFI	TLI	RMSEA (90% CI)	SRMR
Model 1. Unidimensional model	329.379	20	0.790	0.705	0.159 (0.144–0.174)	0.074
Model 2. Correlated two-factor model	324.317	19	0.792	0.694	0.162 (0.147–0.178)	0.074
Model 3. Bifactor model	Did not converge	—	—	—	—	—
Model 4. Re-specified unidimensional model *(Item 1 ~~ Item 2)*	44.150	19	0.983	0.975	0.046 (0.029–0.065)	0.022

*χ*^2^ = chi-square statistic; df = degrees of freedom; CFI = Comparative fit index, TLI = Tucker–Lewis index; RMSEA = Root mean square error of approximation; SRMR = Standardized root mean square residual.

The standardized factor loadings for the final re-specified unidimensional EUROHIS-QOL model are presented in Table 4. All factor loadings were statistically significant (*p* < 0.001), ranging from 0.343 to 0.888. The strongest loadings were observed for the items related to daily functioning, self-satisfaction, interpersonal relationships, and living conditions. In contrast, the global evaluative items assessing overall quality of life and satisfaction with health showed comparatively lower loadings, although they remained significant indicators of the latent construct. The residual covariance added between these two items was statistically significant (standardized covariance = 0.506, *p* < 0.001), supporting the final re-specified model.

**Table 4:** Standardized factor loadings of the final EUROHIS-QOL model in university students.

Item	*λ* (Standardized)	*SE*	*p*-value	95% IC
Item 1	0.392	0.041	<0.001	0.312–0.471
Item 2	0.343	0.046	<0.001	0.253–0.433
Item 3	0.811	0.018	<0.001	0.775–0.847
Item 4	0.789	0.020	<0.001	0.749–0.828
Item 5	0.886	0.016	<0.001	0.855–0.917
Item 6	0.888	0.014	<0.001	0.860–0.916
Item 7	0.855	0.018	<0.001	0.820–0.889
Item 8	0.850	0.018	<0.001	0.814–0.886

λ = standardized factor loading; SE = standard error; 95% CI = 95% confidence interval; p = statistical significance of the factor loadings.

### 3.4 Reliability and convergent validity

The re-specified unidimensional EUROHIS-QOL model demonstrated excellent internal consistency reliability. Ordinal alpha (*α*), McDonald’s omega (*ω*), and composite reliability (CR) coefficients were all above the recommended threshold of 0.70, with values of *α* = 0.907, *ω* = 0.901, and CR = 0.908, respectively. These findings indicate a high degree of internal consistency among the eight items.

In addition, convergent validity was supported by the Average Variance Extracted (AVE), which reached a value of 0.606, exceeding the recommended criterion of 0.50. This result suggests that the latent construct explained a substantial proportion of the variance in its indicators.

### 3.5 Measurement invariance

Measurement invariance of the re-specified unidimensional EUROHIS-QOL model was examined across gender, comorbidity status, and working status using multigroup CFA. Across all grouping variables, the configural, metric, and scalar models demonstrated acceptable to excellent fit indices (Table 5). Changes in CFI and RMSEA between nested models remained within recommended thresholds, supporting full measurement invariance. These findings indicate that the EUROHIS-QOL measures the latent quality of life construct equivalently across the evaluated groups.

**Table 5:** Measurement invariance of the EUROHIS-QOL across groups.

Group	Invariance model	*χ*^2^ (df)	Δ*χ*^2^ (Δdf)	CFI	ΔCFI	RMSEA	ΔRMSEA
Gender	Configural	66.49 (38)	–	0.980	–	0.050	–
	Metric	49.16 (45)	−17.33 (7)	0.987	+0.007	0.007	−0.033
	Scalar	49.65 (53)	0.49 (8)	0.987	0.000	0.007	0.000
Comorbidity status	Configural	53.02 (38)	–	0.999	–	0.026	–
	Metric	53.45 (45)	0.43 (7)	0.995	−0.004	0.025	−0.001
	Scalar	53.45 (53)	0.00 (8)	0.996	+0.001	0.021	−0.004
Working status	Configural	54.47 (38)	–	0.989	–	0.048	–
	Metric	45.26 (45)	−9.21 (7)	0.999	+0.010	0.016	−0.032
	Scalar	46.24 (53)	0.98 (8)	0.999	0.000	0.009	−0.007

*χ*^2^ = chi-square statistic; df = degrees of freedom; Δ*χ*^2^ = chi-square difference; CFI = Comparative Fit Index; ΔCFI = change in Comparative Fit Index; RMSEA = Root Mean Square Error of Approximation; ΔRMSEA = change in RMSEA. Evidence of measurement invariance was supported based on ΔCFI < 0.010 and ΔRMSEA < 0.015.

After establishing scalar invariance, latent mean differences were examined across gender, comorbidity status, and working status. No statistically significant differences were observed between groups. Specifically, latent quality of life scores did not differ significantly between male and female students (*β* = −0.008, *p* = 0.802, 95% CI [−0.069, 0.053]), between students with and without comorbidities (*β* = −0.123, *p* = 0.321, 95% CI [−0.367, 0.120]), or between working and non-working students (*β* = 0.081, *p* = 0.366, 95% CI [−0.095, 0.257]). These findings suggest comparable latent quality of life levels across the evaluated groups.

## 4 Discussion

This study evaluated the psychometric performance of the EUROHIS-QOL in Colombian university students and provides evidence supporting its use as a brief measure of quality of life in a Latin American higher education context. Overall, the findings demonstrated satisfactory reliability, adequate convergent evidence, and measurement invariance across relevant sociodemographic groups. Although the originally specified unidimensional model showed inadequate fit, a theoretically justified re-specification resulted in a parsimonious model with excellent psychometric performance, supporting an essentially unidimensional representation after theoretically justified model refinement.

A central finding of this study concerns the factorial structure of the EUROHIS-QOL. The original unidimensional specification, consistent with the conceptualization proposed by Schmidt et al. (17), did not adequately reproduce the observed covariance structure. Alternative factorial structures were therefore explored, including a correlated two-factor model and a bifactor model. The correlated two-factor model also failed to demonstrate adequate fit, suggesting limited support for a multidimensional interpretation in this sample. The bifactor model failed to converge, suggesting limited empirical support for the coexistence of a dominant general factor and stable domain-specific dimensions in this sample. In contrast, the re-specified unidimensional model—including a residual covariance between the items assessing overall quality of life and satisfaction with health—showed excellent fit indices and retained conceptual interpretability.

The residual covariance between these two items appears theoretically plausible and consistent with the conceptual foundations of subjective quality of life. Both indicators represent broad evaluative judgments regarding overall quality of life and perceived health status and may therefore share variance beyond the common latent construct. This interpretation is also consistent with the conceptual development of the WHOQOL instruments, in which the items assessing overall quality of life and general satisfaction with health are treated separately from the domain structure of the longer WHOQOL and WHOQOL-BREF, functioning as global evaluative indicators rather than domain-specific components (1, 13). Similar residual associations between conceptually overlapping indicators have been observed in psychometric studies of quality-of-life instruments, particularly when brief measures include highly global items (17, 20). Thus, rather than indicating multidimensionality, the present findings support the interpretation of the EUROHIS-QOL as an essentially unidimensional measure in which global evaluative items retain additional shared variance due to conceptual proximity.

These findings align partially with previous evidence (17), in the original cross-cultural validation study, supported the use of a brief global quality-of-life measure with adequate psychometric performance across countries. Likewise, da Rocha et al. (20) demonstrated satisfactory reliability and structural validity of the EUROHIS-QOL relative to its parent WHOQOL-BREF, supporting its utility as a parsimonious alternative for epidemiological and population-based research. However, the present findings also resonate with more recent evidence indicating that alternative structural solutions may emerge depending on the cultural context and sample characteristics. For example, a Brazilian study identified a correlated two-factor solution distinguishing functional and socioeconomic aspects of quality of life, highlighting the possibility that the latent organization of quality-of-life indicators may vary across populations (19). Nevertheless, in the present study, the re-specified unidimensional model demonstrated superior conceptual parsimony and empirical stability, supporting its preference for use in university populations.

The reliability findings further support the adequacy of the instrument. Internal consistency indicators were high, with both ordinal alpha and McDonald’s omega exceeding recommended thresholds, suggesting strong coherence among the eight items. Similarly, average variance extracted and composite reliability supported adequate construct representation and reliability of the latent factor. Together, these findings reinforce previous reports showing that the EUROHIS-QOL provides psychometrically robust measurement despite its brevity (17, 20). From a practical perspective, this brevity may be especially advantageous in university settings, where questionnaire burden represents an important methodological consideration and multiple constructs are frequently assessed simultaneously.

One of the most relevant contributions of this study concerns measurement invariance. Evidence of configural, metric, and scalar invariance was supported across gender, comorbidity status and employment status, indicating that the factorial structure, factor loadings, and item thresholds remained comparable across groups. These findings are particularly important because they suggest that the EUROHIS-QOL assesses quality of life equivalently across relevant subgroups, enabling meaningful comparisons of latent scores without substantial measurement bias (35).

The evidence of invariance according to comorbidity status deserves particular attention. The EUROHIS-QOL has been applied in both general population samples and individuals with physical or mental health conditions, given its purpose as a generic quality-of-life measure (17, 18, 20). Demonstrating scalar invariance between students with and without comorbidities suggests that the instrument retains equivalent measurement properties regardless of underlying health conditions, supporting the comparability of quality-of-life scores across groups with different health profiles. This is especially relevant in university settings, where physical and mental health problems may differentially influence wellbeing and perceived functioning, and strengthens confidence that observed differences reflect substantive variation in quality of life rather than measurement artifacts.

Notably, despite evidence of scalar invariance, latent mean comparisons revealed no statistically significant differences across gender, comorbidity status, or working status. Although previous studies have reported poorer quality of life among students with adverse health conditions or higher academic burden (8), the absence of latent differences observed here may reflect relative homogeneity in the study population or shared contextual influences related to university life. Importantly, these findings should not be interpreted as evidence of true equivalence in lived experiences but rather as indicating that, within this sample, observed differences were not sufficiently large to produce statistically meaningful variation in the latent construct.

Beyond the psychometric findings, the descriptive results provide relevant insights into students’ perceived quality of life. Although most EUROHIS-QOL items reflected moderate levels of wellbeing, lower mean scores were observed in domains related to energy for daily life, financial resources, and daily functioning. These findings are consistent with previous research indicating that university students frequently experience challenges associated with academic demands, financial pressures, and lifestyle changes that may negatively affect their wellbeing (7, 8). From a public health perspective, routine assessment of quality of life in university settings may contribute to the early identification of vulnerable groups and inform the development of health promotion initiatives. Given the increasing recognition of universities as important settings for health promotion, brief and psychometrically robust instruments such as the EUROHIS-QOL may facilitate population monitoring and support evidence-informed interventions aimed at improving student wellbeing.

The study has several strengths. First, it evaluated competing factorial models rather than assuming the adequacy of the original structure, allowing a more rigorous examination of dimensionality. Second, the analysis incorporated multiple psychometric properties—including structural validity, reliability, convergent evidence, measurement invariance, and latent mean comparisons—following COSMIN recommendations for measurement evaluation. Third, the use of a relatively large sample and proportional stratified sampling enhanced representativeness across academic groups.

Some limitations should nevertheless be acknowledged. First, the study was conducted in a single private university in Colombia, which may limit the generalizability of the findings to students from public institutions, other academic disciplines, or educational systems characterized by different social, economic, and organizational contexts. Although participants were recruited from a large private university characterized by disciplinary and socioeconomic diversity, the findings should be interpreted within this institutional context. Future studies should examine the performance of the EUROHIS-QOL across public and private universities in different regions of Latin America to strengthen the external validity of the evidence.

Second, all information was collected through self-report measures, which may introduce response bias, including recall bias and social desirability effects. As quality of life reflects a subjective evaluation of wellbeing, participants’ responses may have been influenced by temporary emotional states, contextual stressors, or individual interpretation of questionnaire items.

Third, although the findings supported an essentially unidimensional structure of the EUROHIS-QOL, the need to incorporate a theoretically justified residual covariance between the global evaluative items assessing overall quality of life and satisfaction with health suggests that the latent organization of the instrument may not be entirely invariant across populations or cultural contexts. The inadequate fit observed for the original unidimensional model and the poor performance of alternative structures indicate that the dimensionality of the EUROHIS-QOL warrants continued psychometric scrutiny, particularly in university populations and Spanish-speaking Latin American settings, where empirical evidence remains scarce.

Finally, the cross-sectional design precluded the assessment of temporal stability and responsiveness of the instrument. Although internal consistency and structural validity were supported, the study did not evaluate test–retest reliability, longitudinal measurement invariance, or sensitivity to change over time. Future longitudinal studies are needed to examine the temporal stability of the EUROHIS-QOL and its ability to capture changes in quality of life during university training or following academic, social, or health-related transitions.

## 5 Conclusion

The EUROHIS-QOL demonstrated satisfactory psychometric performance in Colombian university students, supporting its use as a brief measure of quality of life in higher education settings. The findings support an essentially unidimensional structure with a theoretically justified residual association between global evaluative items, high reliability, and measurement invariance across relevant sociodemographic groups. These results contribute new evidence regarding the applicability of the EUROHIS-QOL in Latin American university populations and support its use in research requiring brief yet psychometrically robust quality-of-life assessment.

## Data Availability

The raw data supporting the conclusions of this article will be made available by the authors, without undue reservation.
